# Guidelines for procedural pain in the newborn

**DOI:** 10.1111/j.1651-2227.2009.01291.x

**Published:** 2009-06

**Authors:** Paola Lago, Elisabetta Garetti, Daniele Merazzi, Luisa Pieragostini, Gina Ancora, Anna Pirelli, Carlo Valerio Bellieni

**Affiliations:** 1 Neonatal Intensive Care Unit, Department of Paediatrics, University of PadovaPadova, Italy; 2 Neonatal Intensive Care Unit, Azienda Ospedaliero-Universitaria-Policlinico di ModenaItaly; 3 Neonatal Intensive Care Unit, Sant' Anna HospitalComo, Italy; 4 Neonatal Intensive Care Unit, San Filippo Neri HospitalRome, Italy; 5 Neonatal Intensive Care Unit, S. Orsola-Malpighi Hospital, University of BolognaBologna, Italy; 6 Neonatal Intensive Care Unit, San Gerardo HospitalMonza, Italy; 7 Department of Paediatrics, Obstetrics and Reproductive Medicine, Policlinico Le Scotte, University of SienaSiena, Italy

**Keywords:** Analgesia, Guidelines, Newborn infant, Pain management, Sedation

## Abstract

Despite accumulating evidence that procedural pain experienced by newborn infants may have acute and even long-term detrimental effects on their subsequent behaviour and neurological outcome, pain control and prevention remain controversial issues. Our aim was to develop guidelines based on evidence and clinical practice for preventing and controlling neonatal procedural pain in the light of the evidence-based recommendations contained in the SIGN classification. A panel of expert neonatologists used systematic review, data synthesis and open discussion to reach a consensus on the level of evidence supported by the literature or customs in clinical practice and to describe a global analgesic management, considering pharmacological, non-pharmacological, behavioural and environmental measures for each invasive procedure. There is strong evidence to support some analgesic measures, e.g. sucrose or breast milk for minor invasive procedures, and combinations of drugs for tracheal intubation. Many other pain control measures used during chest tube placement and removal, screening and treatment for ROP, or for postoperative pain, are still based not on evidence, but on good practice or expert opinions.

Conclusion: These guidelines should help improving the health care professional's awareness of the need to adequately manage procedural pain in neonates, based on the strongest evidence currently available.

## INTRODUCTION


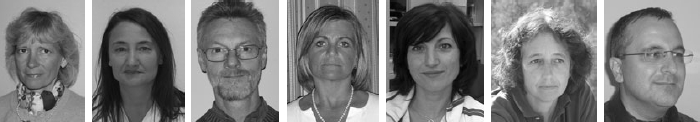


Scientific research in recent years has continued to confirm that neonates, especially when preterm, are more sensitive to nociceptive stimuli than older children. Neonates are capable of mounting robust physiological, behavioural, hormonal and metabolic responses to such stimuli, responses that can have adverse short- and long-term effects (1,2). Several lines of evidence suggest that early and repeated exposure to painful stimuli during a period fundamental to nervous system development leads to persistent behavioural changes and a smaller volume of the sensory areas of the brain in ex-preterm infants (3,4).

The use of pain control for neonates undergoing painful procedures is still limited, however. According to recent reports, neonates at the Intensive Care Unit (NICU) experience a mean 16 painful procedures a day, most of which are still performed without effective pain control measures, as demonstrated by recent surveys (5,6).

The strength of existing evidence is crucial to the quality of pain management. We report here on the clinical guidelines developed by a panel of expert neonatologists who reached a consensus on the recommendations after critically reviewing the latest evidence in 2008.

The present guidelines should help clinicians to choose the most effective and safe pain control measures based on current knowledge. Adequate pain prevention and management should be an essential part of standard health care at the NICU, and recognizing and assessing sources of pain should be routine in the day-to-day practice of physicians and nurses taking care of the newborn.

## METHODS

These guidelines were produced by the Pain Study Group of the Italian Society of Neonatology at four meetings held between June 2007 and January 2008. Each panel member systematically reviewed the literature on a given invasive procedure, evaluating the quality of the data and summarizing the reported pharmacological, non-pharmacological, behavioural and environmental pain control measures in a practical format.

To identify and analyse all available publications on neonatal pain, the Medline (via PubMed), EMBASE and CINHAL databases were searched using *pain, stress, nociception, analgesia, sedation, anaesthesia and premedication, infant, newborn* and *premature* as the key words, and the *Cochrane Library* was checked for critical reviews. The most significant results were presented at meetings of the Study Group experts and discussed until the most appropriate guidelines for each specific procedure were established, based on the strongest evidence drawn from recently published data and/or prevailing clinical practice at NICUs. The method proposed by the Scottish Intercollegiate Guidelines Network (SIGN) was used to draft these guidelines, awarding levels of evidence and grades of recommendation based on the SIGN classification (7) (Table 1). The guidelines were then submitted to critical review by independent professionals operating in various disciplines (paediatricians, anaesthesiologists, pharmacologists, psychologists, nurses) and parents, who also applied an appraisal tool (AGREE) to assess the quality of the clinical guidelines (8). The final draft of the document was approved by the board of the Italian Society of Neonatology in December 2007.

**Table 1:** Grades of recommendation and levels of evidence

Grade of recommendation	Level of evidence
A	At least one good-quality meta-analysis of randomized controlled trials (RCTs) or a sufficiently powered good-quality RCT with a very low risk of bias, directly applicable to the target population
B	A body of evidence including good-quality systematic reviews of case–control or cohort studies directly applicable to the target population, or good-quality case–control or cohort studies with a very low risk of confounders or bias and a high probability of the relationship being causal. Evidence extrapolated from good-quality meta-analyses, systematic reviews of RCTs or RCTs with a very low or low risk of bias
C	A body of evidence including well-conducted case–control or cohort studies with a low risk of confounders or bias and a moderate probability of the relationship not being causal, directly applicable to the target population and demonstrating overall consistency of results, or evidence extrapolated from good-quality systematic reviews of case–control or cohort studies, or good-quality case–control or cohort studies
D	Non-analytical studies, e.g. case reports, case series or evidence extrapolated from well-conducted case–control or cohort studies with a very low risk of bias
Good practice points	Recommended practice, based on the clinical experience of the group that developed the guidelines

Modified from the *SIGN Guidelines Developer's Handbook* 2008.

### Safety considerations

Potential problems deriving from the use of these guidelines relate to the possible side effects of drugs. Knowledge of the pharmacology and pharmacodynamics of the drugs used, which is not within the scope of this document, is essential. Respiratory depression, apnoea leading to bradycardia and desaturation, partial airway obstruction and hypersalivation are the most important side effects of analgesic drugs. However, it is important to emphasize that analgesia and sedation, especially in neonates, should generally be administered by experts capable of immediately recognising and appropriately treating any cardiorespiratory complications. It is therefore important that emergency materials and drugs are always available and readily accessible when stronger drugs are used for analgesia and sedation.

Implementing these guidelines would not incur any additional cost because the drugs involved are already in use at NICUs.

### Editorial independence

These guidelines were developed with no outside funding and the experts on the panel have no conflict of interest with the pharmaceutical industry.

## RESULTS

### General principles

- Environmental, behavioural and non-pharmacological comfort measures are recommended for each procedure, e.g. the use of a pacifier with sucrose combined with distraction techniques (Table 2). The pharmacological options used in combination with these measures can have additive or synergic effects in controlling procedural pain and stress.
  **Table 2:** Environmental, behavioural and non-pharmacological pain control strategies in newborn

	Author	Heel lancing	Veni- puncture	Other
*Sucrose:* in doses from 0.012 to 0.12 g. 12–24% at a dose of 0.2–0.3 mL orally 2 min before the procedure in preterm infants and 1–2 mL in term infants.	Stevens B et al. *Cochrane Rev*. 2004	A	A	–
*Expressed human milk or breastfeeding*	Shah VS et al. *Cochrane Rev*. 2006	A	A	–
*Glucose solution:* dose range 1–2 mL of 10–33% glucose	Skogsdal Y et al.1997 Gradin M et al. 2004 Eriksson M et al.1999 Carbajal R et al. 1999 Carbajal R et al. 1999	C	B	–
*Non-nutritive sucking (NNS)*: i.e. placing a pacifier in an infant's mouth to promote sucking behaviour with no breast or formula milk to provide nourishment	Field T et al. 1984 Shiao Y et al. 1997 Stevens B et al. 1999 Bellieni CV et al. 2001 Corbo MG et al. 2000	B	–	–
*Music therapy:* music with intrauterine sounds or instrumental music in association with NNS	Bo and Callaghan 2000 Butt and Kisilevsky 2000	D	–	–
*Facilitated tucking:* holding the arms and legs in a flexed position	Corff KE et al.1995 Axelin A. et al. 2006 Ward and Larson et al. 2004	C	–	Endotracheal suctioning or routine care C
*Swaddling:* wrapping the neonate in a sheet/blanket	Fearon et al. 1997 Huang et al. 2004 Prasopkittikun and Tilokskulchai 2003 VanSleuwen BE et al. 2007	C	–	–
*Maternal touching and holding:* cradling the baby in the mother's arms	Prasopkittikun and Tilokskulchai 2003	D	–	–
*Kangaroo care or Kangaroo mother care:* the neonate is taken out of the incubator and laid on the mother's or father's bare skin (skin-to-skin contact)	Gray L et al. 2000; Johnston C et al. 2003 and 2008 Ludington-Hoe et al. 2005 Feber Sg et al. 2008	B	–	-
*Positioning: laying the neonate supine*; the evidence of its utility remains inconclusive	Stevens B et al. 1999 Prasopkittikun and Tilokskulchai 2003 Grunau R et al. 2004	–	–	–
*Individualized developmental care*, e.g. limiting environmental stimuli, lateral positioning, using supportive bedding, monitoring behavioural clues, respecting circadian rhythms	Sizun J et al. 2002	–	–	C
*Olfactory stimulation:* vanillin aroma	Goubet et al. 2003	–	C	
*Sensorial saturation:* multiple sensorial stimulation at orogustatory, auditory, olfactory and tactile level	Bellieni et al. 2001	B		–
*Environmental care:* controlling/reducing light and noise	Blackburn 1996, Franck 1998, Brandon 2002, Anand 2001, Menon 1998, Sauve 1995, AAP 1997	–	–	D
*Parental presence during medical procedures*	Axelin A 2006	–	–	D
- For planned procedures, such as blood sampling or creating a vascular access, the optimal baseline state of quiet wakefulness should be obtained before starting the procedure.
- If possible, do not interrupt sleep; plan the procedure far from mealtimes and from any other painful invasive procedures to allow for recovery.
- Conduct the procedure in a calm and relaxing environment, reducing noxious stimuli (light and noise) as much as possible.
- During the procedure, the neonate should preferably be contained in warm sheets and accompanied during and after the procedure.
- Monitoring pain and stress as the fifth vital sign during ongoing analgesia or invasive procedures with scales validated for infants may facilitate the fine tuning of analgesic measures and improve awareness of how the newborn feels.
- At the end of the procedure, continue to monitor the physiological parameters until they return to the baseline state.
- Plan no other invasive procedures for at least 2 h after the procedure.

### Heel lancing

#### Environmental measures

- It is preferable to use venipuncture rather than heel lancing in term neonates and heavier premature infants, since it is less painful and more effective in expert hands [A] (9).
- It is not useful to warm the heel prior to lancing to facilitate blood flow to the area [C] (10,11).
- Use techniques to distract the neonate and provide stimuli to stop pain transmission to the cerebral cortex, such as sensorial saturation (a technique consisting in the mother or nurse massaging and talking to the baby while administering oral glucose before the puncture) [B] (12).
- Consider involving the mother in procedures whenever possible, using skin-to-skin contact or breastfeeding during non-routine sampling [B] (13,14). The efficacy of breastfeeding during multiple painful procedures has not been documented (15).
- Use an automatic lancet of the Tenderfoot variety rather than a manual lancet [B] (16,17).
- Do not squeeze the heel, which must be well perfused and squeezing is itself a cause of pointless pain [D] (18).

#### Non-pharmacological measures

- Use sucrose and non-nutritive sucking (NNS) or human milk [A] (15,19).
- The use of oral sucrose alone has recently proved ineffective in the case of repeated heel lances in term infants in the first 2 days of life [B] (20).
- Alternatively, use a glucose solution [C] (21,22).
- The use of less-concentrated solutions is recommended in premature infants because solutions with higher concentrations of sucrose/glucose (24–33%) have a high osmolarity, up to 1000 mOsm [D] (23).
- The use of NNS seems to have a synergic effect with the sweet taste and is recommended, whenever possible [B] (22,24).
- The use of multiple doses for a given procedure (2 min before, immediately before and 2 min after heel lancing) seems more effective than a single dose [B] (25).
- The long-term safety of multiple doses of oral sucrose has not been demonstrated [A] (26).

#### Pharmacological measures

- The use of EMLA cream is not recommended as it is ineffective for heel lancing pain [B] (27).
- Pre-emptive analgesia with paracetamol before the procedure is not recommended as it is ineffective [A] (28).

### Venipuncture, arterial puncture and percutaneous central venous catheter insertion

#### Environmental measures

Adopt all the *environmental measures* recommended for heel lancing. Choose a smaller gauge trocar cannula of 24–26 G, wherever possible. (This is what the SIGN calls a good practice point [GPP]).

#### Non-pharmacological measures

Using sucrose and NNS or human milk [A] (15,19) seems to be more effective than EMLA [C] (29).

#### Local pharmacological measures

- If it is possible to plan the procedure, apply EMLA cream 60 min beforehand with an occlusive bandage that does not adhere to the skin (to avoid incurring pain on its removal) [B] (27).
- During the application, check for any local reactions (hyperaemia, flushing, areas of cutaneous vasoconstriction) that may occur when a local anaesthetic is applied [D] (30).
- If available, anaesthetics with a faster onset of action (30 min) should be used (liposomal lidocaine 4% cream) [C] (31), while preparations such as tetracaine gel 4% are not to be recommended because they are ineffective in neonates [A] (32).

#### Systemic pharmacological measures

The use of systemic opiate-based analgesia is to be recommended in some situations. In intubated and ventilated neonates, administer a slow i.v. bolus of opiates before the procedure, as necessary [D] (18).

### Intramuscular or subcutaneous injection

#### Environmental measures

It is preferable to administer drugs intravenously wherever possible [GPP].

Adopt all the *environmental measures* mentioned in the heel lancing section. Choose a smaller gauge needle wherever possible [GPP].

#### Local pharmacological measures

Apply EMLA cream (0.5–1 g) 60 min before the procedure [B] (33).

### Central venous catheter insertion by surgical cut-down

#### Non-pharmacological measures

Use sucrose and NNS or human milk during the preparatory phase whenever possible [GPP] (18,19,34,35).

#### Local pharmacological measures

Apply EMLA cream 60 min before the procedure [C] (27) or proceed directly with the subcutaneous infiltration of lidocaine 1% at a dosage of 2–4 mg/kg buffered with sodium bicarbonate (NaCHO_3_ 8,4%) in 1:10 dilution [D] (35). The buffered solution can reduce the pain of the local infiltration (36).

#### Systemic pharmacological measures

- *Sedation*: administer a slow i.v. bolus of fentanyl [D] (18,34,35) and midazolam, as necessary [GPP], or an i.v. bolus of ketamine [GPP].
- Closely monitor the patient and anticipate any need for ventilatory and circulatory support in the event of respiratory depression [GPP].
- General anaesthesia: administer an i.v. bolus of fentanyl and a muscle relaxant [GPP] (Table 3).
  **Table 3:** Analgesic and anaesthetic drugs used in newborn

Drug	Dose	Safety considerations
Local anaesthetic		
EMLA lidocaine–prilocaine 5% cream	0.5–1 g under non-adhesive occlusive dressing 60 min before procedure	Check for any local reactions (hyperaemia, flushing, vaso-constriction) every 15 min
Liposomal lidocaine 4% cream	1 g under occlusive dressing 30 min before procedure	
Lidocaine 1%	2–4 mg/kg buffered with sodium bicarbonate 1:10	Maximum dosage 5 mg/kg
Oxybuprocaine 0.4% and tetracaine 1% eye drops	1 drop per eye	

Systemic analgesic	Bolus dose	Infusion dose

Morphine	50–100 mcg/kg i.v. in 60 min	10–40 mcg/kg/h
Fentanyl	0.5–3 mcg/kg i.v. in 30 min	0.5–3 mcg/kg/h
Acetaminophen or paracetamol	10–15 mg/kg i.v. in 15 min every 6–8 h (i.v.–oral)	

General anaesthetic		

Ketamine	0.5–2 mg/kg i.v.	0.5–1 mg/kg/h
Thiopental	2–6 mg/kg i.v.	
Propofol	2.5 mg/kg	0.5–4 mg/kg/h

Muscle relaxants		

Vecuronium	0.1 mg/kg i.v.	0.05–0.1 mg/kg/h
Mivacurium	0.2–0.3 mg/kg i.v.	

Epidural anaesthetic		

Bupivacaine 0.08–0.1%		0.25 mg/kg/h for max 24–36 h
Ropivacaine	0.9 mg/kg	0.2 mg/kg/h
Levobupivacaine 0.25%	2.5 mg/kg	0.25–0.75 mg/kg/h

### Tracheal intubation

Many different approaches are reported and a great variety of drugs are used, alone or in combination, as premedication for elective intubation in neonates (37). Combinations of opiate and muscle relaxant [B] (38–42) and remifentanil and midazolam [B] (43) or propofol [B] (44), thiopental [B] (45) and ketamine [D] (18) have been proposed. Using appropriate analgesia and sedation during tracheal intubation facilitates the procedure (fewer attempts and shorter times), reducing potentially harmful physiological fluctuations and pain [A] (37). In nasal intubation, small doses (0.3 mL/kg) of lidocaine gel 2% may be useful [D] (46) (Table 4).

**Table 4:** Drug associations for neonatal tracheal intubation

Drugs	Grade of recommendation
Combined i.v. infusions of atropine, an opiate and a muscle relaxant	
Atropine 20 mcg/kg over 1 min + fentanyl 2 mcg/kg over 5 min + mivacurium 200 mcg/kg in rapid infusion	B
Atropine 20 mcg/kg over 1 min + mivacurium 200 mcg/kg over 15–30 sec + fentanyl 5 mcg/kg over 1 min	C
Atropine 20 mcg/kg over 1 min + fentanyl 3–4 mcg/kg over 5 min + suxamethonium 2 mg/kg in rapid infusion	C
Morphine 100 mcg/kg + atropine 10 mcg/kg + suxamethonium 1 mg/kg	B
Propofol 2.5 mg/kg i.v. in a rapid bolus (max 2 doses)	B
Thiopental 6 mg/kg (2.5% solution) i.v. bolus over 1 min	B
Remifentanil 1 mcg/kg over 1 min + midazolam 200 mcg/kg	B
Ketamine 1 mg/kg i.v.+ atropine 20 mcg/kg	D

### Lumbar puncture

#### Environmental measures

Whichever position is chosen (on the side, sitting in the crib or on the nurse's or mother's arm), avoid any extreme flexion of the neck and knees towards the chest because this can cause significant hypoxemia, especially in critical patients (47), as well as carrying a risk of vertebral fractures [D] (48).

It is advisable to perform LP with a G24 Sprotte atraumatic needle, as it separates the fibres of the yellow ligament without severing them, and early stylet removal improves the success rates [C] (49).

This avoids post-LP fluid exudation and the risk of secondary headache and the, albeit rare, onset of epidermoid tumours in the spinal canal, reported after the use of butterfly or other spring needles without stylets [D] (50,51).

#### Non-pharmacological measures

Use sucrose and NNS or human milk [GPP] (15,18,19).

#### Local pharmacological measures

Apply EMLA cream to the puncture site 60 min before the procedure [A] (52).

The use of other local anaesthetics, such as subcutaneous lidocaine infiltration, is not recommended as a front-line anaesthetic measure [C] (53,54), and we have no reports on its use for deeper anaesthesia after EMLA.

#### Systemic pharmacological measures

The use of systemic analgesia and sedation with a slow i.v. opiate bolus can be recommended in some cases if the neonate is intubated. If term infants are not intubated, a bolus of midazolam can be suggested if the infant is particularly restless, monitoring the vital signs (especially blood pressure). After the procedure, keep the neonate supine, continue with pain control measures and monitor the physical parameters until they return to the baseline state [GPP]. Consider using paracetamol for the treatment of headache following subarachnoid puncture [D] (50).

### Chest tube insertion

#### Non-pharmacological measures

Apply appropriate behavioural pain control measures [GPP] (15,18,19,34,35).

#### Local pharmacological measures

If the procedure is not urgent, apply EMLA cream to the puncture site [GPP]. If it is urgent, proceed directly with subcutaneous lidocaine 1% infiltration [D] (18,34,35).

#### Systemic pharmacological measures

In intubated and ventilated neonates, administer a slow i.v. opiate bolus [D] (18,34,35). In non-intubated neonates, consider a bolus of ketamine, except for VLBWI, but anticipate the need for intubation and ventilation in neonates breathing spontaneously [D] (18,35). After the procedure, consider the use of bolus or continuous venous infusions of opiates, monitoring the pain scale [D] (18).

### Chest tube removal

#### Non-pharmacological measures

Apply appropriate behavioural pain control measures [GPP] (15,18,19,34,35).

#### Local pharmacological measures

Apply EMLA cream to the site of insertion [D] (55).

#### Systemic pharmacological measures

Consider a slow i.v. opiate bolus [GPP].

## Screening for ROP

### Non-pharmacological and environmental measures

Perform the screening procedure away from meals [GPP].

Use appropriate behavioural pain control measures and sucrose and NNS [A] (56) or human milk [GPP] (15,19).

Conduct the ophthalmoscopy without using the blepharostate, the positioning of which causes pain [C] (57).

### Pharmacological measures

In the case of RetCam screening, apply local anaesthesia with oxybuprocaine 0.4% or tetracaine 1% eye drops and consider a slow i.v. opiate bolus or ketamine [D] (57,58).

### Laser therapy for ROP

#### Non-pharmacological measures

During preparations for the procedure, adopt appropriate behavioural pain control measures [GPP] (15–19).

#### Pharmacological measures

In general, combine a *local anaesthetic* with a *general anaesthesia*, administering a slow i.v. opiate bolus in association with a muscle relaxant before intubation, or combine *local anaesthesia* with *sedation* using low doses of opiates combined with midazolam or ketamine, supporting the airways with a positive pressure [D] (58). Nasopharyngeal prongs or a laryngeal mask are a valid alternative to ventilatory support during brief measures if the neonate has not already been intubated [D] (59). At the end of the procedure, arrange for postoperative analgesia for the first 24–48 h [D] (18).

## CONCLUSION

These guidelines on analgesia and sedation in neonates undergoing invasive procedures should help improving the health care professional's awareness of the need to adequately manage procedural pain in neonates, based on the strongest evidence currently available. There is a large body of evidence to support the utility of sucrose and NNS for pain prevention during heel lancing and venipuncture, but it is not clear whether multiple doses of sucrose are safe. The role of breastfeeding or breast milk for pain control has also been documented in the last few years. NNS and sensorial saturation are other recommended techniques.

In the case of tracheal intubation, several studies have demonstrated the efficacy of various drug combinations comprising an analgesic (usually an opiate) and a muscle relaxant in reducing pain and facilitating the procedure, though the superiority of any given combination remains to be established. Many other analgesics are commonly used in practice, e.g. for chest tube placement and removal, lumbar puncture, screening for ROP and laser therapy, but their usage is not evidence based.
